# Patterns of frugivory in the columnar cactus *Pilosocereus* *leucocephalus*

**DOI:** 10.1002/ece3.4833

**Published:** 2019-01-08

**Authors:** Shamira Vázquez‐Castillo, Antonio Miranda‐Jácome, Ernesto Ruelas Inzunza

**Affiliations:** ^1^ Facultad de Biología Universidad Veracruzana Xalapa Veracruz Mexico; ^2^ Instituto de Investigaciones Biológicas Universidad Veracruzana Xalapa Veracruz Mexico; ^3^ Instituto de Biotecnología y Ecología Aplicada Universidad Veracruzana Xalapa Veracruz Mexico

**Keywords:** Bird, frugivory networks, mammal, Mexico, Rancho San Ignacio, seed dispersal, Veracruz, Xalapa

## Abstract

In the frugivory networks of many arid and semi‐arid Mesoamerican ecosystems, columnar cacti act as keystone species that produce fruits with a high content of water and nutrients attractive to numerous vertebrates. The aim of this investigation was to assess the fruit removal patterns of two guilds of frugivores on the fruits of the woolly torch *Pilosocereus leucocephalus*. We assessed fruit pulp removal in two ways: by estimating the consumption of seeds given the amount of pulp removed per visit and by estimating the percentage of pulp removal over time. We put exclosures on unripe, intact fruits to keep frugivores from removing material. Once ripe, we removed the exclosures and tracked animal visitation of 69 fruits using camera traps. We obtained a total of 2,162 hr of footage (14:47 hours of them with effective pulp removal). The highest number of visitors is that of diurnal species (*n* = 12, all birds) versus only four nocturnal (three bats, one rodent). The most effective species in pulp removal are birds. Bats play a modest role in frugivory of this cactus. The significance of this work is twofold: (a) birds and bats consume the fruit pulp of this cactus and likely disperse its seeds, and (b) although bats rank high in pulp removal effectiveness, birds as a guild far outweigh their importance in this system, as they are not only more frequent but also remove more pulp and seeds. Both groups are known to be important in cacti seed dispersal, and our findings are essential in understanding the population dynamics of the woolly torch and in elucidating its seed dispersal ecology.

## Resumen

En las redes de frugivoría de muchos ecosistemas áridos y semiáridos mesoamericanos, los cactus columnares funcionan como especies claves que producen frutos con un alto contenido de agua y nutrientes atractivos a numerosos vertebrados. El propósito de esta investigación fue determinar los patrones de remoción de pulpa de los frutos del cactus viejito *Pilosocereus leucocephalus* por dos gremios de frugívoros. Determinamos los patrones de remoción de pulpa de dos maneras: estimando el consumo de semillas dada la cantidad de pulpa removida en cada visita y estimando el porcentaje de pulpa removida por unidad de tiempo. Colocamos jaulas de tela de alambre sobre frutos sin madurar para excluir frugívoros. Una vez maduros los frutos, quitamos las jaulas y registramos las visitas de animales en 69 frutos utilizando cámaras‐trampa. Obtuvimos un total de 2162 h de video (14.47 h de estas con remoción efectiva de frutos). El mayor número de visitantes fue de especies diurnas (n=12, todos aves) vs. solo cuatro nocturnos (tres murciélagos, un roedor). Las especies más efectivas en la remoción de frutos son aves. Los murciélagos juegan un papel modesto en la frugivoría de este cactus. El significado de este trabajo es doble: (1) aves y murciélagos consumen la pulpa de los frutos de este cactus y muy posiblemente dispersan sus semillas, y (2) aunque los murciélagos se ubican entre los visitantes más efectivos en la remoción de pulpa, las aves les aventajan de lejos en importancia en este sistema dado que no solo son más frecuentes, sino que remueven más pulpa y semillas. Ambos grupos son conocidos por su importancia en la dispersión de semillas de cactus y nuestros hallazgos son esenciales en el entendimiento de la dinámica poblacional del cactus viejito y en dilucidar la ecología de su dispersión de semillas.

## 1 INTRODUCTION

### 1.1 Frugivory is a complex ecological process influenced by multiple factors

This relationship between plants and animals and its ecological outcomes are a result of the interplay among environmental conditions, the reproductive strategy of plants, and the role that animals play in seed dispersal (Gomes, Quirino, & Araujo, 2014; Schupp, Jordano, & Gómez, 2010).

The function of columnar cacti in frugivory networks in arid and semi‐arid regions is of paramount importance to our understanding of those ecosystems (Bravo‐Hollis, 1978). In these nutrient‐limited habitats, cacti provide a variety of fruits rich in water and sugars to vertebrates and invertebrates that consume their mucilaginous pulp and presumably help in the dispersal of seeds (Schupp, 1993). One could presuppose the frugivory networks of all groups of columnar cacti are generally similar with fine‐grain differences among them (e.g., Montiel & Montaña, 2000), but before ecologists can engage in solving complex questions related to the economy of costs and benefits in such frugivory networks, it is important to elucidate some basic issues.

We are interested in the population ecology of the woolly torch *Pilosocereus leucocephalus* (Poselg.) Byles & G.D. Rowley as a study system. This Mesoamerican species, as well as many others in all four clades of columnar cacti, has received little attention from researchers in spite of its keystone position in tropical dry ecosystems. Some basics of the role of animals in its frugivory networks are unknown (Munguía‐Rosas & Sosa, 2010), and the first few questions of importance in understanding these relationships include: Which species of animals are frugivores of *P. leucocephalus*? What is the relative importance of each guild/species in removing its fruit pulp? Moreover, how can we appropriately determine the patterns of frugivory by understanding the relative contribution of variables such as the amount of fruit pulp consumed, the number of seeds fed upon, and the duration and frequency of visits? And, ultimately, does frugivory by animals mean seed dispersal, predation, or a mix?

We hypothesized that a quantitative assessment of pulp removal by different animal visitors would help us move beyond basic descriptions and allow us to uncover some of the ecological processes (like the ones listed above) that underlie the frugivory patterns of this cactus, since many aspects of the ecology of fruit‐bearing plants depend largely on which animal species are charged with dispersing its seeds.

This paper provides new information on *P. leucocephalus* visitation by birds and mammals. Here, we estimate foraging time, amount of fruit pulp removal, and quantify the effectiveness of diurnal and nocturnal guilds in removing fruit. We also assess the role of each guild of visitors in seed dispersal and seed predation given what we know about cacti and their dispersal agents.

## 2 MATERIALS AND METHODS

### 2.1 Study site

**Figure 1:**
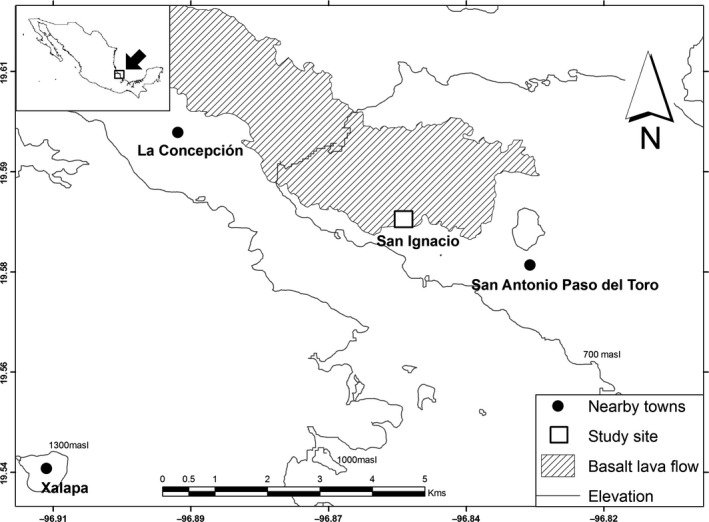
The Rancho San Ignacio, a private estate near the city of Xalapa, Veracruz, Mexico, has a population of the woolly torch (*Pilosocereus leucocephalus*) that grows over an ancient basalt lava flow covered with tropical deciduous forest

We studied the patterns of frugivory of *P. leucocephalus* in a natural population located at the Rancho San Ignacio, near the town of San Antonio Paso del Toro in the Municipality of Xalapa, Veracruz, Mexico (19.8°35′26″N, 96.8°58′38″W, elevation 1,150 m above sea level, hereon masl; Figure 1).

Besides being an accessible study site, the fact that this is a private estate allowed us to have control over human visitation and alterations associated with them such as human traffic modifying the patterns of cactus visitation and the robbery or tampering of camera equipment.

The dominant substrate of Rancho San Ignacio is a Holocene basalt lava flow (locally known as malpaís) about 10,000 years old (Negendank et al., 1985). The mean annual temperature is 24°C and its annual precipitation is 1,300 mm (79.1% of which is concentrated in a rainy season between the months of May‐October, Fernández‐Eguiarte, Romero‐Centeno, & Zavala Hidalgo, 2012). The vegetation of this site is primarily tropical deciduous forest (selva baja caducifolia, Miranda & Hernández Xolocotzi, 1963).

The dominant plant families at this site are Fabaceae, Asteraceae, and Poaceae. A conspicuous element that stands out is the presence of succulent plants of the families Euphorbiaceae, Orchidaceae, Cactaceae, and Bromeliaceae (Castillo‐Campos, 2003).

### 2.2 Natural history of* Pilosocereus leucocephalus*

With 36 species (all of them in the Americas), the genus *Pilosocereus* belongs to the largest of all four tribes of columnar cacti (Godínez‐Alvarez & Valiente‐Banuet, 1998). Our knowledge of frugivory patterns in all these taxa is scarce and lists of visitors to three species of the genus *Pilosocereus* (*P. leucocephalus*, *P. polygonus*, and *P. purpusii*), and a few closely related genera, are the only information available for the entire clade. Three groups of vertebrates, birds, bats, and reptiles are listed in previous studies (Kissling, Böhning‐Gaese, & Jetz, 2009; Naranjo, Rengifo, & Soriano, 2003; Ruiz, Santos, Cavelier, & Soriano, 2000; Silva, 1988; Silvius, 1995; Tschapka, Sperr, Caballero‐Martínez, & Medellín, 2008; Wendelken & Martin, 1988). Beyond those lists of visitors, Soriano et al. (1999) made the only available study that attempts to quantify the relative importance of various frugivore visitors to two columnar cacti in Venezuela.

*P. leucocephalus* is endemic to Mesoamerica. It occupies lowland areas dominated by tropical deciduous forests (from about 250 masl) to the transition with cloud forest (at about 1,150 masl), from central Mexico to Nicaragua (Guzmán, Arias, & Dávila, 2003; Missouri Botanical Garden, 2018).

This cactus exhibits synchronized fruiting pulses from April to September. Its fruit is spheroid in shape, about 6 × 5 cm, and weighs 60–100 g. When ripe, it has a large amount of reddish mucilaginous endocarp (pulp) that contains its seeds (Munguía‐Rosas, Jácome‐Flores, Sosa, & Quiroz‐Cerón, 2009). Seeds are oval, with a mean length of 1.99 mm (±0.19) and a mean width of 1.33 mm (±0.12) (measurements taken from a set of 50 randomly sampled seeds from 15 different fruits, AMJ unpublished data). The number of seeds per fruit is highly variable ($\overset{¯}{x}$ = 1,431 ± 97 [*SE*], range = 265–4,152, *n* = 62 fruits, Rodríguez García, 2016).

The features of its fruit (red and fleshy) suggest zoocoran dispersal. From previous published work, we can infer flying vertebrates do most of its dispersal. In their study based on extensive mistnetting of visitors, Munguía‐Rosas et al. (2009) report nine species of birds and four of bats feeding on *P. leucocephalus* fruits and point out that two genera of ants do remove seeds from the ground and could be additional agents of seed dispersal.

### 2.3 Selection of cactus individuals

During the April‐September 2015 fruiting season, we selected 100 individuals of *P. leucocephalus* along a linear transect. The plants selected had to be 1.5 m high or taller to ensure they were part of the reproductive population. Fruit is typically placed in the upper half to upper third of the cactus. All individuals selected for this study were numbered and marked with aluminum tags. From those, we performed camera trap recordings on 69 cacti that produced fruits during our field season.

To ensure the complete development of cactus fruits and the complete filming of its consumption by animals, we placed poultry wire exclosure cages ~15 cm in diameter by 15 cm depth on each of the fruits under study. Exclosure cages were attached with wire to the main stem of the cactus. Cages protected the fruits against frugivores for about 30 days (the estimated time that it takes to ripen). We removed the exclosures once fruits were ready (when its shape and color were those of a ripe fruit) and began tracking visitors with camera traps.

### 2.4 Animal visitor records

We used eight camera traps to identify bird and mammal visitors (Supporting information S1), but given the sparse seasonal distribution of fruiting in *Pilosocereus*, the highest number of cameras simultaneously recording frugivory was four. To understand the daily patterns of frugivory, we divided the daily cycle into two periods. The two intervals, from 7:00–19:00 and from 19:00 to 7:00 hours, recorded two different animal guilds foraging on *P. leucocephalus* fruits (hereon we call them “diurnal” and “nocturnal” guilds, respectively). We recorded frugivory on all studied fruits from start (100% of the fruit) until final consumption (0% of the fruit left).

**Figure 2:**
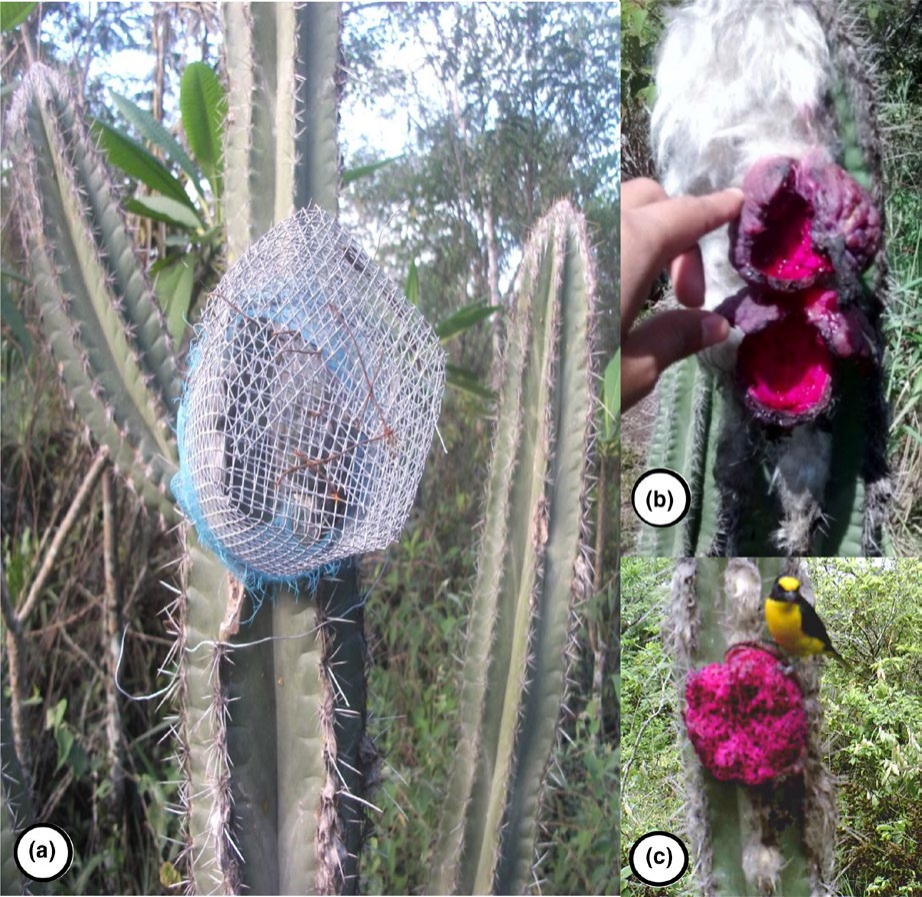
A photo mosaic illustrates the set‐up of camera traps to track patterns of frugivory in *Pilosocereus leucocephalus*. (a) Exclosure cage on an individual of *P. leucocephalus*, (b) Ripe, focal fruit, and (c) Ripe fruit with a frugivore visitor (a yellow‐throated euphonia, *Euphonia hirundinacea*)

We identified visitors by analyzing stills and video footage from camera traps. For birds, we used the field guides by Howell and Webb (1995) and Sibley (2003). Mammals were identified using Álvarez‐Castañeda, Álvarez, and González (2015), with bat identification aided with Munguía‐Rosas et al. (2009). The analysis of each video consisted of recording in a datasheet the start and end time of each frugivore visit. After each visit, we estimated the amount of fruit consumed by comparing photos taken before and after each visit (overlaying a 10 × 10 cm grid on each photo) and estimating a percentage of removal (Figure 2).

### 2.5 Measures of frugivore effectiveness

We estimated frugivore effectiveness in two different ways: (a) Quantifying the proportion of a fruit's pulp removed over time, with an index described below and (b) Calculating the estimated consumption of seeds per visit.

For the first type of estimate, we devised an index to measure the amount of fruit removed per unit of time. For this, we gave the value of 100% to intact fruits and 0% to fruits empty of pulp. This index is a quotient of the estimated percentage of fruit mucilage (pulp and seeds included) removed over time over the number of individuals of each species or guild:

$$\text{IER}=\frac{\sum \left(\frac{q_{\text{i}}-q_{\text{f}}}{t_{\text{f}}-t_{\text{i}}}\right)}{n}$$

Where *q*_i_ = initial quantity of pulp, *q*_f_ = final quantity of pulp, *t*_f_ = final visit time, *t*_i_ = initial visit time, and *n* = number of recorded observations per frugivore species or per guild. Because this index is a quotient, the highest values are those of the most effective frugivores, whereas those values approaching zero suggest a very ineffective removal of fruit pulp.

We also calculated Estimated Seed Consumption (ESC), an estimation of the total amount of seeds removed per visit. For this, we obtained 62 ripe fruits of *Pilosocereus* and removed all the pulp from them. For each fruit, we counted its number of seeds, that ranged between 265–4,152 ($\overset{¯}{x}$ **= 1,431, *SE* = 97), and used the percentage of pulp removed to calculate ESC.

We compared the performance between species and guilds using ANOVA. The fixed factor in these analyses was the guild (with two levels) with IER or ESC values as a response variable. At the species level, the fixed factor was the species (with 14 levels) and the response variable the IER or ESC. Because IER values for species and guilds were non‐normal, we transformed them and used a Wilcoxon signed ranks tests (Conover & Iman, 1981). For each of these two analyses, we conducted their respective Tukey post hoc tests.

To understand the daily cycle pattern of frugivory, we took the start time of visitation from the camera trap display and converted them to degrees in order to use Rayleigh's test of circular statistics (that demonstrates the uniformity of records given its angle). Species that have an aggregated pattern over time can be singled out below its significance threshold, whereas those that do not forage at different times of the day in a non‐aggregated fashion. Degree values are then back transformed to time of the day and plotted as the mean in a circular diagram.

## 3 RESULTS

### 3.1 The diversity of *P. leucocephalus* visitors

We obtained a total of 2,162 hr of footage on 69 different fruits of *P. leucocephalus*. From this material, 14:47 hours (0.64% of the time) pertained to foraging activity. We identified 15 species of birds and mammals on 213 foraging events on *P. leucocephalus* fruits. The majority of this foraging activity took place during the night‐time period (11:35 hours), an almost fourfold difference over daytime fruit pulp removal (3:11 hours). If we exclude the single mouse species recorded (four visits, see below), the total chiropteran visitation time is dramatically reduced to 0.68 min, meaning that diurnal visitors spend >280× more time foraging in *Pilosocereus* fruits than flying nocturnal visitors.

Species recorded in night‐time foraging (Table 1) are three species of bats: Pallas's long‐tongued bat, (*Glossophaga soricina*), Godman's long‐tailed bat (*Choeroniscus godmani*), and Sowell's short‐tailed bat (*Carollia sowelli*), as well as a single rodent: Mexican deer mouse (*Peromyscus mexicanus*). Because of the difficulty of separating the three species of bats of individual foraging events, we lumped them into a single category for analysis.

**Table 1:** Species (and species groups) foraging activity on the fruits of the columnar cactus *Pilosocereus leucocephalus* in Veracruz, Mexico

Species or group	*N* of visits	Foraging time (min) ($\overset{¯}{x}$ ± SE)	% of Fruit consumption ($\overset{¯}{x}$ ± SE)	ESC (per visit) ($\overset{¯}{x}$ ± SE)	ESC % (cum total)	IER ($\overset{¯}{x}$ ± SE)
*Icterus galbula* □*N*	63	2.02 ± 0.41	3.52 ± 0.71	451 ± 177	21.4% (3,160)	3.76 ± 0.60
*Campylorhynchus rufinucha* □	43	0.25 ± 0.04	3.83 ± 0.59	157 ± 45	16.01 (2,359)	22.26 ± 2.66
Bats ●	34	0.02 ± 0.01	0.52 ± 0.02	45 ± 20	1.84 (271)	47.25 ± 1.91
*Passerina ciris* (Pr) □*N*	14	0.83 ± 0.29	1.07 ± 0.21	35 ± 9	1.45 (214)	5.74 ± 2.05
*Psilorhinus morio morio* □*N*	13	0.27 ± 0.04	20.0 ± 3.16	572 ± 171	27.18 (4,004)	83.99 ± 11.9
*Euphonia hirundinacea* □*N*	13	0.52 ± 0.20	0.96 ± 0.14	35 ± 13	1.21 (178)	11.15 ± 2.54
*Melanerpes aurifrons* □	7	0.51 ± 0.35	7.14 ± 2.16	143 ± 68	4.85 (715)	24.04 ± 5.38
*Psilorhinus morio fuliginosus* □*N*	6	0.59 ± 0.21	20.8 ± 2.19	314 ± 28	10.68 (1,573)	56.23 ± 13.5
*Chlorophonia occipitalis* □*N*	5	0.07 ± 0.02	0.8 ± 0.13	19 ± 6	0.39 (57)	13.00 ± 2.26
*Euphonia affinis* □*N*	4	0.31 ± 0.31	1.75 ± 1.25	na	0.68 (100)	14.01 ± 4.43
*Peromyscus mexicanus* ●*N*	4	172.5 ± 127.2	35.5 ± 22.52	1,015 ± 185	13.78 (2,030)	1.18 ± 0.74
*Icteria virens* □*N*	3	0.06 ± 0.91	1.0 ± 0.0	na	0.29 (42)	17.26 ± 3.90
*Amazilia tzacatl* □*N*	3	0.04 ± 0.03	0.5 ± 0.0	na	0.14 (21)	0.50 ± 0.00
*Passerina versicolor* □	1	0.59 ± 0.00	0.65 ± 0.21	na	0.05 (7)	1.10 ± 0.25

Notes

We obtained these records throughout the fruiting phenology period April‐September 2015. □ = Diurnal, ● = Nocturnal. Foraging time is expressed in min (mean ± standard error [*SE*]) and fruit consumption expressed as the percentage of endocarp (mucilaginous pulp) removed from fruits (mean ± *SE*). IER: index of effective removal; ESC: estimated seed consumption (mean ± *SE*, total). Two species recorded as visitors deserve special mentions for their protection status or rarity (*Passerina ciris*, Pr = special protection status, and *Chlorophonia occipitalis*, a cloud forest specialist, very rare in this part of its range). *N* = new report of frugivory on *P. leucocephalus*. Species listed by frequency of visits.

The diurnal guild is composed of 12 species of birds (including two distinctive forms/subspecies of one of them, the brown jay, *Psilorhinus morio*) (Table 1).

The diurnal guild of frugivores had a much higher number of visits (*n* = 175 visits). Among these, two species stand out for the frequency of its foraging bouts, Baltimore oriole (*Icterus galbula*) and rufous‐naped wren (*Campylorhynchus rufinucha*), with 63 and 43 visits respectively. The nocturnal guild of frugivores has fewer visits (*n* = 38), and bat species account for the largest number of foraging events (34 visits).

The duration of foraging events is highly variable but tends to be short (<1 min). Mexican deer mouse, for example, took the longest to forage, with an average visit time of over 3 hr, whereas the varied bunting (*Passerina versicolor*), had a single visit of <1 min. Bats make notoriously quick visits, the shortest of all visitors (Table 1).

### 3.2 Fruit pulp removal estimated with an index

While bird diversity plays the key role in frequency of visits (82% of the total number of visits), the difference in foraging effectiveness between diurnal and nocturnal guilds showed a mixed pattern. Nocturnal visitors (18% of the total number of visits) rank high among the most effective species in fruit pulp removal per unit of time than diurnal ones (IER mean values of 42.40 ± 2.89 vs. 18.13 ± 2.05, respectively). The difference in mean effectiveness is statistically significant (*F*_1,212_ = 27.75, *p ≤ *0.0001).

The role of the single mouse species’ four visits plays a key role in this estimate. The four mice visits account for >99% of nocturnal guild frugivory time. When eliminated from the tally, bats are reduced to a miniscule proportion of the total foraging time, although their effectiveness is high (Table 1).

**Figure 3:**
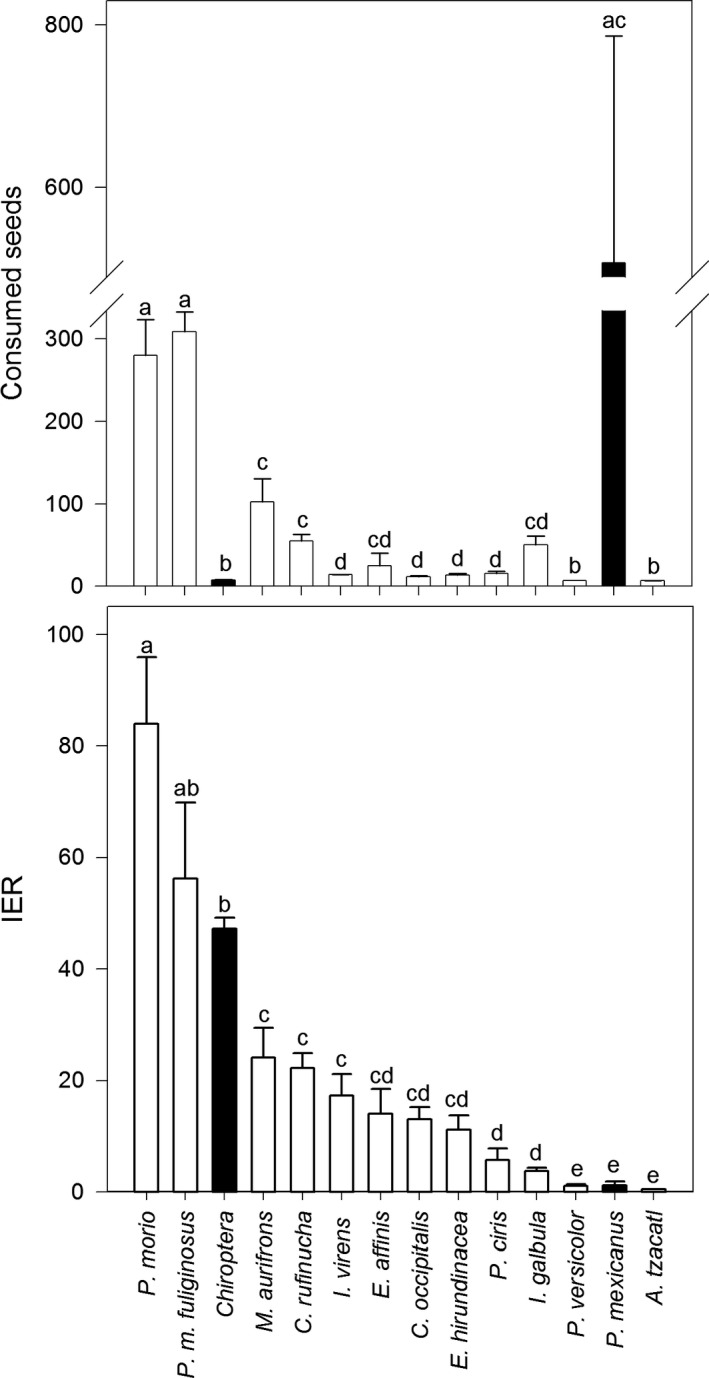
Estimated (a) fruit pulp removal and (b) removal effectiveness of diurnal and nocturnal visitors of *Pilosocereus leucocephalus*. The letters above each bar represent groupings of species/guilds with similar (e.g., undetected in orthogonal comparison) pulp removal (a) and IER values (b). Comparisons yield four and five separate groupings, see text for further details

We also documented differences in foraging effectiveness among species. At one end of the spectrum, the most effective species is the brown jay (specifically the form *Psilorhinus morio morio*, with an IER value of 83.99 ± 11.9), and the least effective is the rufous‐tailed hummingbird *Amazilia tzacatl* (IER = 00.5 ± 00.00). These differences are also statistically significant (*F*_13, 200_ = 34.11, *p* ≤ 0.0001, Figure 3).

### 3.3 Estimated seed consumption

Nearly 85% the total ESC is done by birds (the remaining percentage, done by mammals, is reduced to <2% when including only bats, Table 1). Three species dominate ESC, brown jay, Baltimore oriole, and rufous‐naped wren. Birds are very different from each other in both their total ESC and their ESC by visit (Table 1, Figure 3).

### 3.4 Time of visits

**Figure 4:**
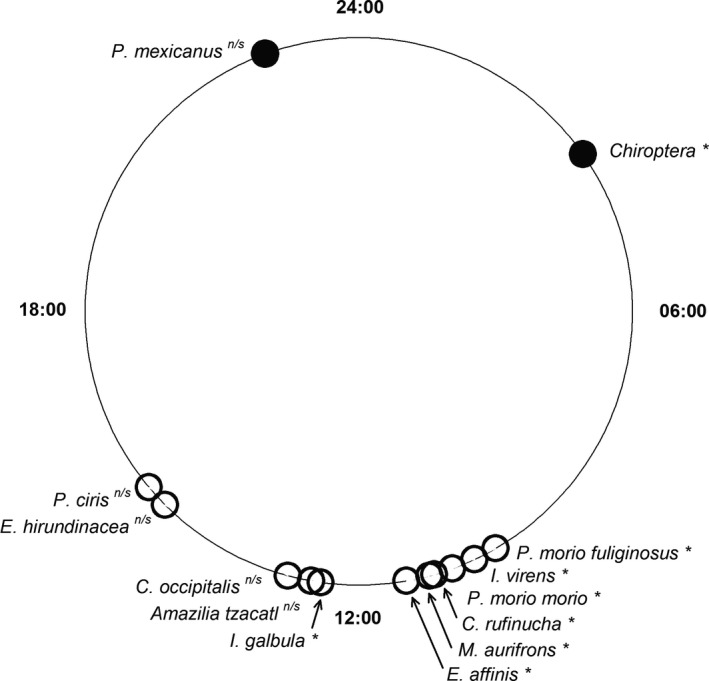
Time of fruit visitation by diurnal and nocturnal frugivores in the columnar cactus *Pilosocereus leucocephalus*. Species/groups marked with an asterisk are those with statistically significant aggregation times using Rayleigh's test

Eight out of 13 species/groups with more than one fruit visit show an aggregated pattern of frugivory over time (Figure 4; Supporting information Table S2). From species with aggregated frugivory visits, the time of visitation of mice happens before midnight, whereas that of bats occurs right before sunrise. Birds in turn forage from late morning to slightly past noon (Figure 4; Supporting information Table S2).

## 4 DISCUSSION

### 4.1 Frugivore diversity

Most of the species feeding on the fruits of woolly torch had never been recorded as frugivores of *Pilosocereus* and very few reports overall document lists of frugivores visiting columnar cacti (Table 1). Wendelken and Martin (1988) reported three species in common with our study (golden‐fronted woodpecker, *Melanerpes aurifrons*, rufous‐naped wren, and varied bunting, *Passerina versicolor*) in their investigation in the tropical dry forests of Guatemala. In spite of the large distance between Wendelken and Martin's study site and that of our work, and the potentially large pool of species that could be feeding on this cactus, it is remarkable that our investigations coincide in these species. The rufous‐naped wren stands out among these—it is the second most frequently recorded visitor in our report and one that also plays an important role in Wendelken and Martin's (1988) study. The correspondence between these small sets of interacting species separated by a large geographic distance suggests this is a frugivory network of a certain degree of specialization (Dáttilo & Rico‐Gray, 2018, Dehling 2018).

The most frequent visitor (one of the three most effective in removing fruit and one of the top species in ESC, Table 1) of *P. leucocephalus* is a migratory species, the Baltimore oriole. This bird's seasonal visit only overlaps with part of the fruiting season of *P. leucocephalus* (this oriole is present in the area approximately from mid‐September to mid‐April, Rising & Flood, 1998) and may undertake longer‐distance movements than most other visitors. It would be worthwhile assessing its role in seed dispersal. Our work contrasts with work done with other species that may be dispersed over much shorter distances (for example, *Melocactus schatzlii*), a species where the fruit of this globular cactus are not consumed by birds and mammals but by lizards (*Ameiva provitae* and *Cnemidoporus lemniscatus*, Casado & Soriano, 2010).

The role of bats in this system is by far secondary to that of birds. Bats are effective in removing fruit material and might be of importance to *P. leucocephalus* given what is known about the role of bats in the dispersal and germination of seeds (Olea‐Wagner, Lorenzo, Naranjo, Ortiz, & León‐Paniagua, 2007). The fact that the peak abundance of fruits coincides with our highest number of records of chiropteran visitation gives additional support to the idea of synchrony between these two events. Furthermore, the three species of bats recorded in our study are known to disperse cacti seeds (Naranjo et al., 2003).

Novoa, Cadenillas, and Pacheco (2011) recorded Pallas's long‐tongued bats feeding on *P. leucocephalus* in a separate study. *Glossophaga* bats might be key in cactus seed dispersal; Soriano et al. (1999) examined the food habits of Miller's long‐tongued bats (*G. longirostris*) and found seeds of pitayo de mayo (*Stenocereus griseus*), hedge cactus (*Subpilocereus* [*Cereus*] *repandus*), and another torch species (*Pilosocereus tillianus* [syn.: *Cephalocereus moritzianus*]). Tschapka et al. (2008) also published a list of cacti fruit consumed by bats.

Our results show the fruits of *P. leucocephalus* are a frequently used food resource for vertebrate frugivores. It supports what has been described in the literature (e.g., Bravo‐Hollis 1978) that the fruit of *P. leucocephalus*, a red, fleshy berry, would be primarily consumed by a diurnal guild of frugivores. These fruits are more readily consumed by birds than by mammals (it takes three times more time for nocturnal visitors to consume an equivalent amount of pulp). Our data also suggest birds may play a more central role in the seed dispersal and population dynamics of this cactus than mammals. Moreover, the shape and color of the fruits of *P. leucocephalus*, and the higher species richness in the diurnal guild, suggests they have been selected to be dispersed primarily by birds (Soriano et al., 1999).

The nocturnal guild, however, also plays an important role in this system. Our data suggests that bats can be as effective (although not nearly as frequent, resulting in a low ESC) as frugivore birds. The role of mammals in seed dispersal has been less widely studied than that of birds (Frick, Price, Heady, & Kay, 2013), and its study with camera traps may help uncover key information on their role in this system.

In spite of an abundant body of literature studying plant‐frugivore relationships, not much is known about the role of the non‐flying mammal fauna in the predation, dispersal, and recruitment of cactus seeds. Columnar cacti, for example, are assumed to have thorny stems as a natural barrier to keep rodents and other small mammals from reaching its fruit. Non‐flying mammals are broadly known to be seed predators rather than dispersal agents (Sosa & Fleming, 2002).

One recent study evaluated the effect of carnivores on seed dispersal and germination, and reported that seed consumption by these animals might increase the probability of germination after passing through its digestive tract (Morales‐Paredes, Valdivia, & Sade, 2015). Our observations of the single rodent visitor and several studies (Heithaus, 1981; Janzen, 1971) indicate this may not be the case in our study system. The chewing and cracking of *P. leucocephalus* seeds by Mexican deer mouse indicates the damage varies from severe to complete destruction (Campos & Vélez, 2015). Other reports agree with this statement: rodents, when compared to birds or bats, are regarded as significantly poor in the dispersal and recruitment of seeds (Janzen, 1971).

The interaction of the two guilds of frugivores with *P. leucocephalus* may range from seed predation to mutualism depending on the animal species (Fedriani & Suárez‐Esteban, 2015). In cacti, recent studies demonstrate that frugivore mammals are the main agent of seed dispersal (Escribano‐Ávila, Pías, Escudero, & Virgós, 2015). Our results concur to some extent with this idea. Excluding the aforementioned rodent, our footage shows bats are effective frugivores that consume a large amount of fruit pulp in a short amount of time and deposit visually intact seeds away from the source plant (Naranjo et al., 2003).

### 4.2 Frugivore pulp removal over time and in consuming seeds

Very few studies have explored the effectiveness of frugivores on the dispersal of seeds. Campos and Vélez (2015) addressed this issue in their study and highlight the fact that frugivore effectiveness depends on the sites where those seeds are deposited and whether those places can favor germination and the probability of establishing new individuals in a population. Rojas‐Robles, Stiles, and Muñoz‐Saba (2012) point out that frugivore effectiveness is positively related to a larger consumption of fruit seeds, as well as distance travelled and final destination of transported material. Our work does not address the location of final deposition of seeds, but considers effectiveness as a function of the number of seeds consumed, the frequency of fruit visits, and the amount of fruit pulp and seeds consumed over time.

The only paper we know that uses an index to determine frugivore effectiveness is that of Soriano et al. (1999). Their approach is slightly different to ours: these authors estimate seed consumption based on visit time and number of seeds consumed over time. However, they made experiments under laboratory conditions where fruit were offered to 19 species of captive birds associated with two cacti (*Stenocereus griseus* and *Subpilocereus repandus*).

Soriano et al.’s (1999) paper states the number of seeds consumed per unit of time is the best tool for estimating the importance of a frugivore in a community. Our investigation builds on this finding, except we estimate consumption times directly in the field, under natural conditions, using camera traps.

Our research indicates that most of the visitors and most of the feeding events on the fruit of *Pilosocereus leucocephalus* may effectively be seed dispersal events, with a proportion of those visits actually being predation by mice. Frugivory research on columnar cacti should move beyond documenting interactions and focus on quantifying patterns and evaluating the differential role of each species or group of visitors on the dispersal of fruits. The outcome of our investigation foresees four issues that warrant further investigation. (a) A comprehensive evaluation of the role of seed passage through the digestive tract of birds and bats: Is the germination and establishment of *P. leucocephalus* seedlings positively or negatively related to consumption? (b) Do mice prey upon *Pilosocereus* seeds? (c) Studies of the role of tree canopy and nearby vegetation in the frugivory of *P. leucocephalus*: Does nearby shelter increase visitation and effectiveness of frugivory? and (d) Determining the differential effectiveness of the two co‐occurring forms of the brown jay in the population dynamics of this little‐known cactus: Why does frugivory by two forms of the same bird species varies so greatly?

## CONFLICT OF INTEREST

None declared.

## AUTHOR CONTRIBUTIONS

All authors revised the manuscript and approved the final version.

## Data Availability

Data available from the Dryad Digital Repository: https://doi.org/10.5061/dryad.26c0m59.
