# Supplementary material for: Patterns of frugivory in the columnar cactus Pilosocereus leucocephalus
Source: Ecol Evol. 2019 Jan 8;9(3):1268–77. doi: 10.1002/ece3.4833 (PMC6374677; doi:10.1002/ece3.4833)
Supplement: Supplementary file 1 [file ECE3-9-1268-s001.docx]

**Supplementary information**

**S1**

**Camera trap use in the study of frugivory in columnar cacti**

Our paper reports the first known use of these widely used devices to study frugivory in cacti (O’Connell *et al*. 2011, Frick et al. 2013). In our study, assessing these patterns of *Pilosocereus leucocephalus* required a precise quantification of frugivore visits. The technology and application of camera traps to study ecological processes has seen great advances in recent years (O’Connell *et al*. 2011). Camera traps could also eliminate human-induced effects of previous observational research, such as limited capabilities for recording, identifying, and estimating time and amounts of fruit removed by visitors (particularly those foraging at night), biases that may result from observer presence, labor-intensive mistnetting of and manipulation of individuals that inhibits normal behaviour of visitors, and others. An important part of this investigation was centred on overcoming the limitations found in observational and mistnetting studies.

Camera trap technology has made substantial advances in recent years (especially shutter speed, quick-response motion sensors, and black light flashlights), and can track date, time, and consumption time in diurnal and nocturnal frugivores, and can also be used to quantify removal patterns with a higher accuracy. Because camera traps can shoot stills and video, footage analysis can be of great help in identifying species as well as accurately estimating foraging bouts. Cameras are motion activated and collect video while the visitor is moving.

We used Wildview STC-WV24 camera traps (manufactured by Wildview Stealth Cam LLC, Grand Prairie TX 75053) affixed to a cactus stem about 2 m of the focal fruit. Diurnal shooting was done without a flash and nocturnal one using only infrared light to avoid chasing away frugivores. Camera traps were placed in discrete sites facing the fruits and in the best possible site to allow high quality images of visitors. Up to four cameras collected data simultaneously. Video footage was stored in 32 Mb, class 10, SanDisk Extreme SDHC cards (Western Digital Technologies, Inc., Milpitas CA 95035) and later transferred onto a computer hard drive for analysis.

The quality of frugivore’s footage, however, deserves stating that daytime camera data has a higher resolution that allows an easier identification of visitors at the species level. Night-time camera data, in turn, has some limitations. It can appropriately quantify the fruit pulp removal patterns, but species identification is a lot more complex. We suspect that in species-rich systems, the proper identification of visitors may be compromised and limit the identifications to a family level (providing there is a good knowledge of the local pool of possible species).

Camera traps were essential in identifying all species, and this was particularly important for nocturnal visitors that are hard to identify by sight. Camera traps have seen a surge in studies of wildlife, but apparently not for understanding frugivory (O’Connell *et al*. 2011, O’ Brien & Kinnaird 2008). We did not test the differences between observer and camera-trap data in this paper, but it is our impression that camera-trap data are less prone to methodological noise (e.g. interobserver differences, fatigue, or uneven identification skills) and that any biases that may be a consequence of the use of this technique are systematic and could be fixed in further studies.

Table S2. Time of the day and number of bird and mammal visits to fruits of the columnar cactus *Pilosocereus leucocephalus* in Veracruz, Mexico. *U* values are those of Reynolds circular statistics text, where aggregated records are denoted statistically significant with an asterisk and those that are distributed broadly over time are not. Species are arranged by frequency of visits; we excluded single visits from this analysis.

| Species | N visits | *U* | *P* |
| --- | --- | --- | --- |
| *Icterus galbula* | 63 | 4.471 | * |
| *Campylorhynchus rufinucha* | 43 | 7.078 | * |
| Chiroptera | 34 | 5.951 | * |
| *Passerina ciris* | 14 | 0.394 | ns |
| *Psilorhinus morio morio* | 13 | 3.501 | * |
| *Euphonia hirundinacea* | 13 | 0.001 | ns |
| *Melanerpes aurifrons* | 7 | 2.656 | * |
| *Psilorhinus morio fuliginosus* | 6 | 3.051 | * |
| *Chlorophonia occipitalis* | 5 | 1.414 | ns |
| *Euphonia affinis* | 4 | 2.068 | * |
| *Peromyscus mexicanus* | 4 | 1.331 | ns |
| *Icteria virens* | 3 | 2.232 | * |
| *Amazilia tzacatl* | 3 | 1.331 | ns |
